# Characterization and Identification of NPK Stress in Rice Using Terrestrial Hyperspectral Images

**DOI:** 10.34133/plantphenomics.0197

**Published:** 2024-07-24

**Authors:** Jinfeng Wang, Yuhang Chu, Guoqing Chen, Minyi Zhao, Jizhuang Wu, Ritao Qu, Zhentao Wang

**Affiliations:** ^1^ College of Engineering, Northeast Agricultural University, Harbin 150000, China.; ^2^ Yantai Agricultural Technology Popularization Center, Yantai 261400, China.; ^3^ College of Life Sciences, Northwest A&F University, Yangling 712100, China.

## Abstract

Due to nutrient stress, which is an important constraint to the development of the global agricultural sector, it is now vital to timely evaluate plant health. Remote sensing technology, especially hyperspectral imaging technology, has evolved from spectral response modes to pattern recognition and vegetation monitoring. This study established a hyperspectral library of 14 NPK (nitrogen, phosphorus, potassium) nutrient stress conditions in rice. The terrestrial hyperspectral camera (SPECIM-IQ) collected 420 rice stress images and extracted as well as analyzed representative spectral reflectance curves under 14 stress modes. The canopy spectral profile characteristics, vegetation index, and principal component analysis demonstrated the differences in rice under different nutrient stresses. A transformer-based deep learning network SHCFTT (SuperPCA-HybridSN-CBAM-Feature tokenization transformer) was established for identifying nutrient stress patterns from hyperspectral images while being compared with classic support vector machines, 1D-CNN (1D-Convolutional Neural Network), and 3D-CNN. The total accuracy of the SHCFTT model under different modeling strategies and different years ranged from 93.92% to 100%, indicating the positive effect of the proposed method on improving the accuracy of identifying nutrient stress in rice.

## Introduction

Among the world’s most important field crops, rice contributes to global high-quality development, hunger reduction, and environmental variety [1]. This topic has undergone thorough investigation over an extended period of time [2–5]. However, the quality of the above services depends on the yield and quality of rice, but crops often suffer from various biotic and abiotic factors during their growth process [6]. Stress has emerged as a markedly limiting factor affecting rice production and quality because of the detrimental effects of various stresses (biotic and abiotic stress) on crop growth [7]. Among these, nutrient stress, such as NPK stress, can lower yield and crop failure while causing corresponding changes in the external morphology, biochemical component content, photosynthesis, enzyme activity, and meristem function in rice plants [8–10]. Too low NPK content will lead to slow plant growth, resulting in rice yield reduction [11], while excessive application of nutrients will lead to overgrowth of plants, prone to lodging, and increase the risk of pests and diseases [12]. In addition, nutrient stress may also lead to a decline in soil quality, further affecting soil conservation capacity and ecological function [13]. A dependable approach that can track minute variations in crop growth, physiology, and production factors is needed to quantify heterogeneity quickly and avoid detrimental effects on crop yield and the environment. This approach should also be able to pinpoint the primary reasons of these variations, in order to maximize crop management and resource investment in the future.

The most widely utilized techniques for tracking crop stress are still field research and physicochemical analysis [14]. Despite its high reliability, the former has subjective, labor-intensive, and time-consuming problems. The latter monitors crop stress with high accuracy, but it has problems such as cumbersome steps, expensive equipment, and destructive testing. The patterns of plant nutrient stress can be efficiently observed using remote sensing technology. Previous research has shown that hyperspectral data from space [15] and ground-based platforms [16] can be widely used for non-biotic stress assessment and vegetation monitoring. Furthermore, spectral response pattern variations can be used to infer the variability of the plant health index [17], which includes biodiversity [18–21], non-structural carbohydrates, leaf area index, and canopy nitrogen concentration. While some recently launched satellites are able to provide sub-meter ground resolution [22], other factors that may have an impact on the accuracy of aviation and aerospace image retrieval include the satellite’s altitude, complex atmospheric conditions, highly mixed farmland communities, and systematic errors related to orbit angle and solar radiation pressure on the Chinese BeiDou Navigation Satellite System, Geostationary Orbit, and Inclined Geostationary Orbit satellites [23]. This implies that flaws frequently go undetected until they have already propagated widely. Drones, however, are able to offer a ground sample distance (GSD) of less than 1 cm. However, as Barbedo sums up the state of drone research and application, this technology also has some drawbacks [24]. Specialized training is necessary for drone operation, there may be stringent flight regulations, crash accidents frequently occur, and meteorological variables can cause impaired picture resolution or potential flight obstruction. Therefore, using in situ or ground spectroscopy for close range image measurement is a comparatively feasible option to achieve high spatial resolution.

Complexity abounds in hyperspectral imaging (HSI). Extracting relevant information from a vast amount of data is the most challenging aspect of the HSI application [25]. In order to demonstrate the association between spectral input and plant nutrient stress modes, previous research has effectively employed machine learning methods, including support vector machines (SVMs), random forest, and multiway partial least squares analysis [26–28]. However, Chen et al.’s study [29] compared the stacked autoencoder with logistic regression and SVM algorithms, and found that the model’s performance is determined by preprocessing technology and professional experience. Zhang et al. [30] used Savitzky–Golay smoothing, the derivative transformation, standard normal variate, and 3 other methods to analyze the spectra of winter wheat samples, and found that the preprocessing methods vary depending on the specific tasks. There is a pressing requirement to create a comprehensive analytic method that can extract characteristics from the original graph and enhance the model’s capacity to generalize. Deep learning has emerged as a substitute technique in recent years for improving performance in classification and regression issues because of its feature learning capability [31]. Deep learning for classification can obtain the same or even superior calibration performance compared to conventional machine learning techniques, according to studies by Audebert et al. [32] and Yang et al. [33]. Deep learning technology’s Transformer architecture has garnered attention lately. Vaswani et al. [34] have successfully applied transformer architecture to practical tasks. Transformer introduction has been the subject of numerous studies aimed at improving HSI task solutions. He et al. [35] directly employed the transformer network for HSI classification and proposed the HSI-bidirectional encoder representations from the transformer (BERT). In order to capture spectral spatial characteristics and advanced semantic features, Sun et al. [36] integrated CNN and Transformer. Currently available advanced approaches are not as good as the suggested spectral spatial feature tokenization transformer method for classification performance. The above research proves that the combination of CNN and Transformer structure will help to analyze the sample characteristics more fully and can markedly improve the classification performance, which has become a hot technical topic for its advantages. Unfortunately, to our knowledge, research on deep learning combined with hyperspectral remote sensing technology to identify rice NPK stress has not yet been reported. On the other hand, there is a limited number of studies investigating the impact of N, P, and K stress on the spectral reflectance of rice leaves [37,38]. The possibility of using spectral information to simultaneously evaluate the effects of N, P, and K states and their interactions in physiological responses and vegetation indices still needs to be completely investigated. Insufficient information in this area can result in misinterpretation when assessing the health condition of rice plants and hinder the decision-making processes related to it.

Considering the above background, this study intended to identify different stress modes of NPK in rice while using terrestrial hyperspectral images and deep learning. Our work objectives were as follows: (a) conducting field experiments to establish and obtain rice HSI images under different nutrient stresses; (b) using spectral profile, unsupervised visualization, and vegetation index to indirectly characterize rice under different nutrient stresses; (c) proposing an SHCFTT model based on CNN and Transformer architecture, which creatively combines unsupervised feature extraction module, a spectral–spatial feature extraction module, a Gaussian weighted feature tokenizer, and a transformer encoder (TE) module, developing a deep learning classification network for identifying nutrient stress patterns in rice; and (d) analyzing the classification results of the built deep learning model under different modeling strategies in different years and evaluating the performance of the model while comparing with the recognition results of classical SVM, 1D-CNN, and 3D-CNN.

## Materials and Methods

### Experimental site and design

**Fig. 1.**
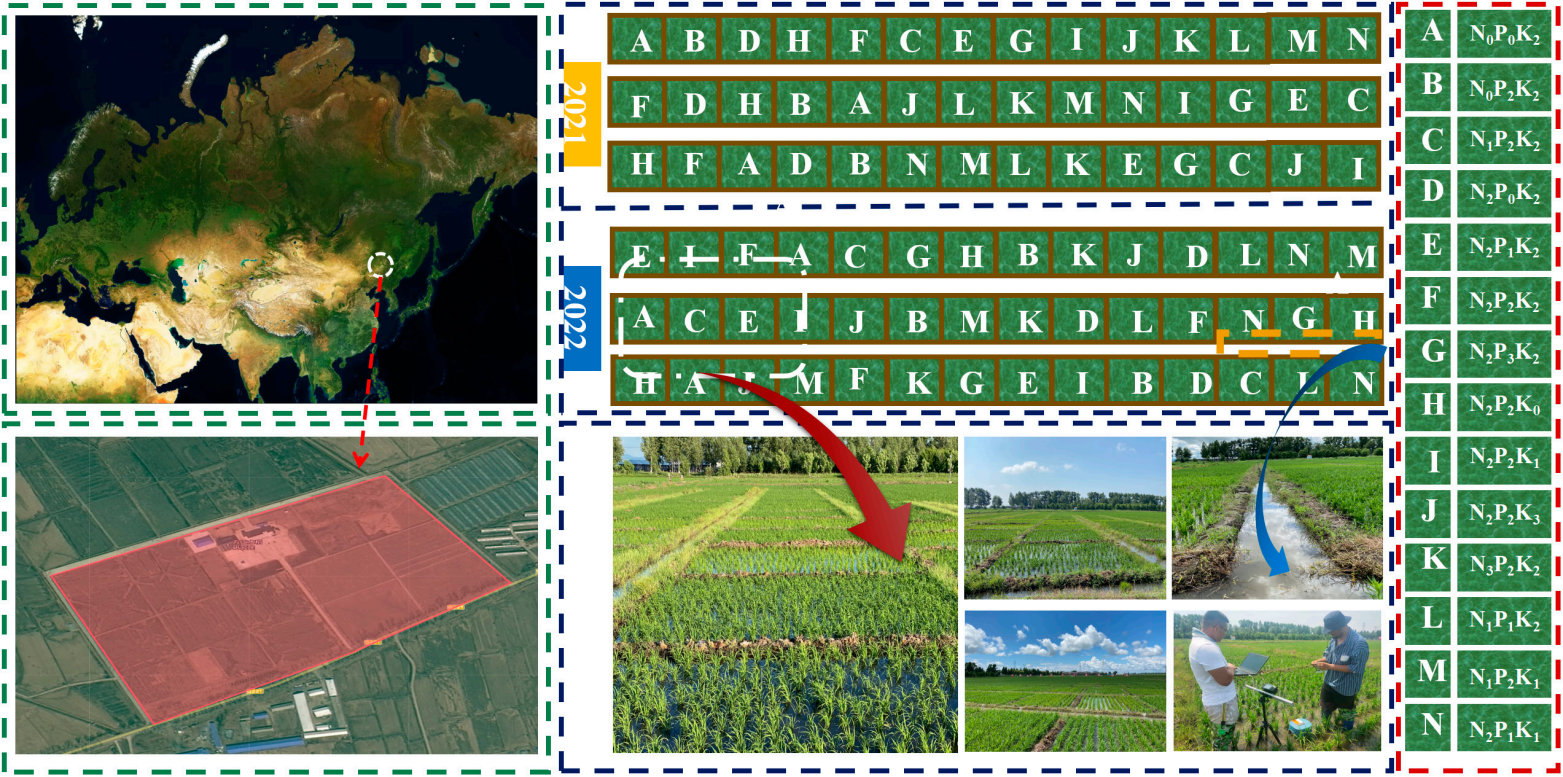
Experimental location and fertilization test block arrangement.

The experiment was conducted for 2 years (2021 to 2022) at the Rice Research Base of Fangzheng County, Heilongjiang Province, China, at an altitude of 124 ± 0.7 m (east longitude 128°13′41″ to 129°33′20″, north latitude 45°32′46″ to 46°09′00″). This region falls within the cold temperate continental monsoon climatic zone, with an average annual temperature of 3.8 °C. The mean annual precipitation is 579.7 mm, while the mean annual sunlight duration is 4,446 h. The soil samples taken from the experimental field at a depth of 0 to 20 cm were analyzed for their nutrient composition, and the results showed that they contain an organic matter content of 42.01 g/kg, an alkali-hydrolyzed nitrogen content of 73.68 mg/kg, an available phosphorus content of 54.43 mg/kg, and an available potassium content of 136.9 mg/kg. Aromatic Rice No. 2 (135-day growth period, active accumulated temperature 2,550 °C, 13-leaf variety) was chosen as the experimental rice variety. According to the quadratic regression D-optimal design, the NPK stress experiment was divided into 14 treatments, each with 3 replicates. The experimental sites were divided into 42 small communities, each with an area of 24 m^2^. The specific arrangement is shown in Fig. 1. All rice plants were transplanted through manual transplanting on 2021 May 16 and 2022 May 20, respectively. Four levels of N, P, and K nutrient stress were produced using urea, heavy superphosphate calcium, and potassium chloride with 2 levels being the local average fertilization amount obtained through investigation and survey. The whole plot treatment included N_0_ (extreme), N_1_ (low), N_2_ (normal), and N_3_ (high) N rates (i.e., 0, 5, 10, and 15 kg/666.67 m^2^, purity quantity); P_0_ (extreme), P_1_ (low), P_2_ (normal), and P_3_ (high) P rates (i.e., 0, 1.5, 3, and 4.5 kg/666.67 m^2^, purity quantity); and K_0_ (extreme), K_1_ (low), K_2_ (normal), and K_3_ (high) K rates (i.e., 0, 3, 6, and 9 kg/666.67 m^2^, purity quantity); this is shown on the right side of Fig. 1. The fertilization ratio corresponding to each stage of rice is 50% nitrogen + 100 phosphorus + 50% potassium for base fertilizer, 20% nitrogen for striking root fertilizer, and 30% nitrogen + 50% potassium for tillering fertilizer. During the whole period of plant growth, it is important to maintain the height and density of all plots at a level that effectively prevents water seepage, water overflow, and the inflow and outflow of nutrients from neighboring plots. Uniform implementation of weed management and disease prevention techniques is necessary across all tiny plots, in accordance with their specific requirements.

### Spectrum collection

To reduce air interference and capture detailed spectral data for rice nutrient stress studies, we purchased a state-of-the-art hyperspectral camera known as SPECIM IQ (model 0604675, Olu, Finland). The SPECIM IQ incorporates functionalities, such as the collection, examination, manipulation, and visualization of hyperspectral data. The SPECIM IQ camera was operated using the SPECIM IQ Studio software and connected remotely using either USB or Wi-Fi for control. The remote control enables all camera functions with the exception of focusing. SPECIM IQ is capable of capturing comprehensive hyperspectral photos without the need for external movement or experiencing any slight delays in recording distinct sections of the image, whether spatially or spectrally. The hyperspectral image acquired by the camera was a 3D dataset with 204 bands, 512×512 pixels spatial sampling and a 31° by 31° field view. It can capture a target from a minimum distance of 150 mm to an infinite distance. For a more comprehensive understanding of this camera, refer to the research conducted by Wang et al. [39].

This study utilizes the Labsphere Spectralon panel as a reference during field measurements to ensure consistent data collection throughout various different conditions and diurnal variations. The panel is chosen for its nearly perfect reflectance of 99% within the 250- to 2500-nm band. The factor of utmost importance for capturing hyperspectral images of nutritional stress in rice is the variation in irradiance resulting from the fluctuations in solar intensity and atmospheric conditions. To mitigate potential atmospheric effects, it is advisable to minimize the time lapse between the reference and target measurements, considering that the majority of reflectance measurements are currently conducted using a single field of view. This optimization is also crucial because it must ensure the consistency of illumination feature for the reference and target measurements. Furthermore, it is necessary to take into account the adjustment for the non-Lambertian behavior of the standard when using any field reflectance standard.

The reference measurement can be calibrated using Eq. 1 in the following manner if any field standard *ρ*_*λ*s_ is incorporated into the process:

$$\rho {\left(\lambda \right)}_{\text{corr}}=\Phi_{\lambda r,t_{1}}\frac{\rho_{\lambda s}}{\Phi_{\lambda i,t_{0}}}$$ (1)

In this scenario, *ρ*(*λ*)_corr_ represents the reflectance of the spectrum adjusted for non-Lambertian field reference and *ρ*_*λ*s_ represents the spectrum reflectance factor of the field standard. Φ_*λ*r_ and Φ_*λ*i_ represent the reflected spectral radiant flux and incident spectral radiant flux, respectively. Because in most circumstances, *t*_0_ − *t*_1_ ≠ 0 and Δ*t* < 15min, *ρ*_*λ*s_ needs to be applied to Φ_*λ*i_ before multiplying it with the reflected radiance. Sunphotometer observations may be used to quantify the stability of the atmosphere at certain wavelengths.

**Fig. 2.**
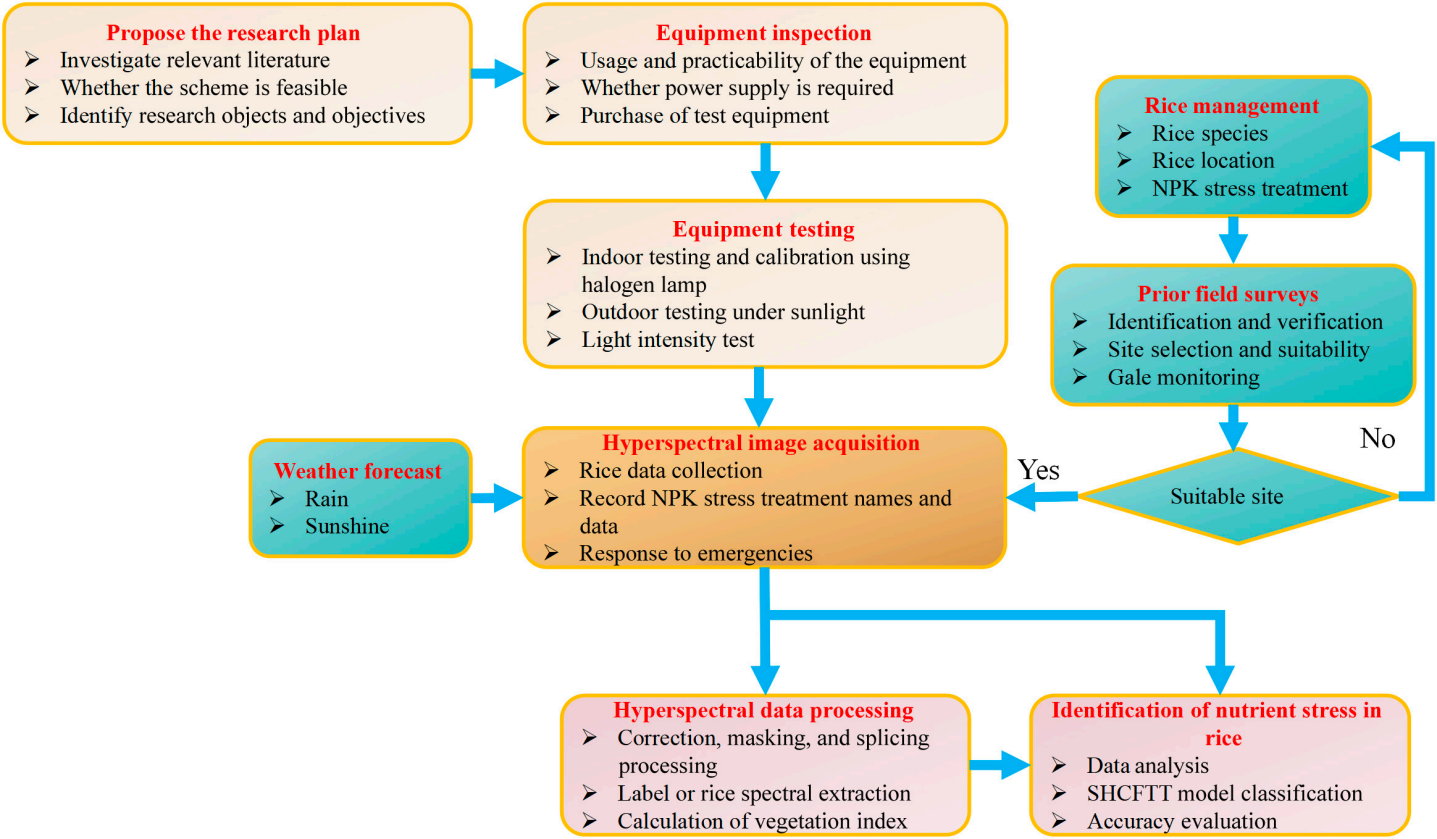
The flow diagram of the study.

After completing fertilization treatments in 2021 and 2022, field information acquisition started on the seventh day. As shown in Fig. 1 (bottom right), a hyperspectral camera was placed about 70 cm above the tested object. A total of 420 (5×14×3×2) hyperspectral images of 14 NPK nutrient stresses were collected with a data size of approximately 1.4 TB. The vast majority of captured hyperspectral images include background regions, which must be excluded before calculating the 14 NPK nutrient stress features and deep learning recognition classification. Therefore, ENVI was applied to each hyperspectral image to create a binary mask layer that could mask the background area including water, Labsphere Spectralon panel, and soil. Typical masking processing is shown in Fig. S1. Figure 2 shows the detailed flow of the distribution program for obtaining and analyzing hyperspectral images from a ground-based hyperspectral camera. In addition, deep-learning classification models require label making on hyperspectral images. To guarantee that the sample labels are of a high quality, manual labeling was exclusively used. The analysis and processing of SVM, 1D-CNN, spectral profiles, principal component analysis (PCA), etc. require obtaining representative spectral information from the sample. The extraction method references Wang et al.’s [40] research. For the atmospheric correction, image splicing, and ROI (region of interest) extraction in this study, the ENVI 5.4 software (Research Systems Inc., CO, USA) was used. The label making was accomplished using the open-source tool labelme in Python 3.7 (Python Software Foundation, Wilmington, DE, USA).

### Method

#### Spectral diversity of NPK nutrient stress

To illustrate the variations in spectral reflectance of rice caused by different nutrient stressors and how the spectral reflectance of stress samples and NPK are related, PCA of spectral reflectance of rice stress samples from various spectral regions can be extracted and use PCA to analyze the spectral variety of rice under various nutrient stresses [41,42]. The variation in the rice stress samples’ spectrum reflectance across different spectral regions is indicated by the amplitude of the PC coefficient. These differences are linked to the biochemical and structural characteristics of rice as well as how it interacts with NPK. Therefore, the PC’s shifting pattern throughout the wavelength reveals how the stress level and NPK affect the reflectance of the rice canopy. Furthermore, the results of the PCA may indicate variations in stress kinds and levels that are present in different types of rice and offer the interpretability of the rice stress modal identification technique.

#### Vegetation index

When crop growth is under stress, corresponding physiological structures, pigments, and enzymes will undergo changes. These substances contain rich chemical bonds, which are strongly absorbed in the visible and near-infrared (NIR) regions. These intense vibration bands form recognizable bands in the spectral region [43]. The vegetation indices (VIs) that are produced could offer effective and straightforward methods for mapping crop stress, health status, and structure in both qualitative and quantitative domains. Empirical and semi-empirical spectral indices, such as the Normalized Difference Vegetation Index (NDVI), Photochemical Reflectance Index (PRI), and Plant Sensitivity Reflectance Index (PSRI), are examples of classic vegetation indices [44]. NDVI is the most extensively used VI. It is formed from plant’s converse spectral response patterns in the red and NIR regions of the electromagnetic spectrum. It is highly correlated with vegetation greenness, health condition, and vegetation productivity [45]. Since mesophyll collapse occurs before chlorophyll decline, NDVI serves as an early indicator of plant stress before visible changes in stress occur.

$$\text{NDVI}=\frac{\rho_{\text{NIR}}-\rho_{\text{RED}}}{\rho_{\text{NIR}}+\rho_{\text{RED}}}$$ (2)

To mitigate the changes related to the sun’s diurnal angle, the PRI’s applicability was also investigated to determine the variations of nutrient stress in 14 types of rice. PRI is a photosynthetic index based on narrowband reflectance, obtained by normalizing the difference in reflectance around the 531- and 570-nm wavelength regions. It is thought to have great potential in directly estimating net photosynthetic rate [46]. Net photosynthetic rate is an important indicator to measure photosynthesis capacity as well as vegetation productivity, which could reflect the overall growth of plants. The structure and functional state of plant leaves improve with increasing net photosynthetic rate. The PRI is particularly related to the conversion of carotenoids in the lutein cycle, which is crucial for preventing excessive light exposure [47].

$$\text{PRI}=\frac{\rho_{531}-\rho_{570}}{\rho_{531}+\rho_{570}}$$ (3)

Different crop phenotypes might create distinct spectral characteristics according to the PSRI, which is especially sensitive to the aging stages of the crop. Crop productivity, yield analysis, and vegetation canopy stress monitoring can all be done with it [48].

$$\text{PSRI}=\frac{\left(\rho_{680}-\rho_{500}\right)}{\rho_{750}}$$ (4)

#### T-distributed stochastic neighbor embedding

A nonlinear, unsupervised, manifold-based FE technique called t-distributed stochastic neighbor embedding (t-SNE) uses cluster analysis and dimensional reduction to visualize data [49]. It is being used to handle HSI data and allows for an overview of complex multivariate data. t-SNE provides an understanding of the spatial organization of the data in a high-dimensional space. In this case, t-SNE helps visualize high-dimensional data while preserving its essential structure. The dataset can be utilized for exploration because it gives a precise description of the complete dataset.

#### Deep learning algorithm SHCFTT for rice NPK stress identification

**Fig. 3.**
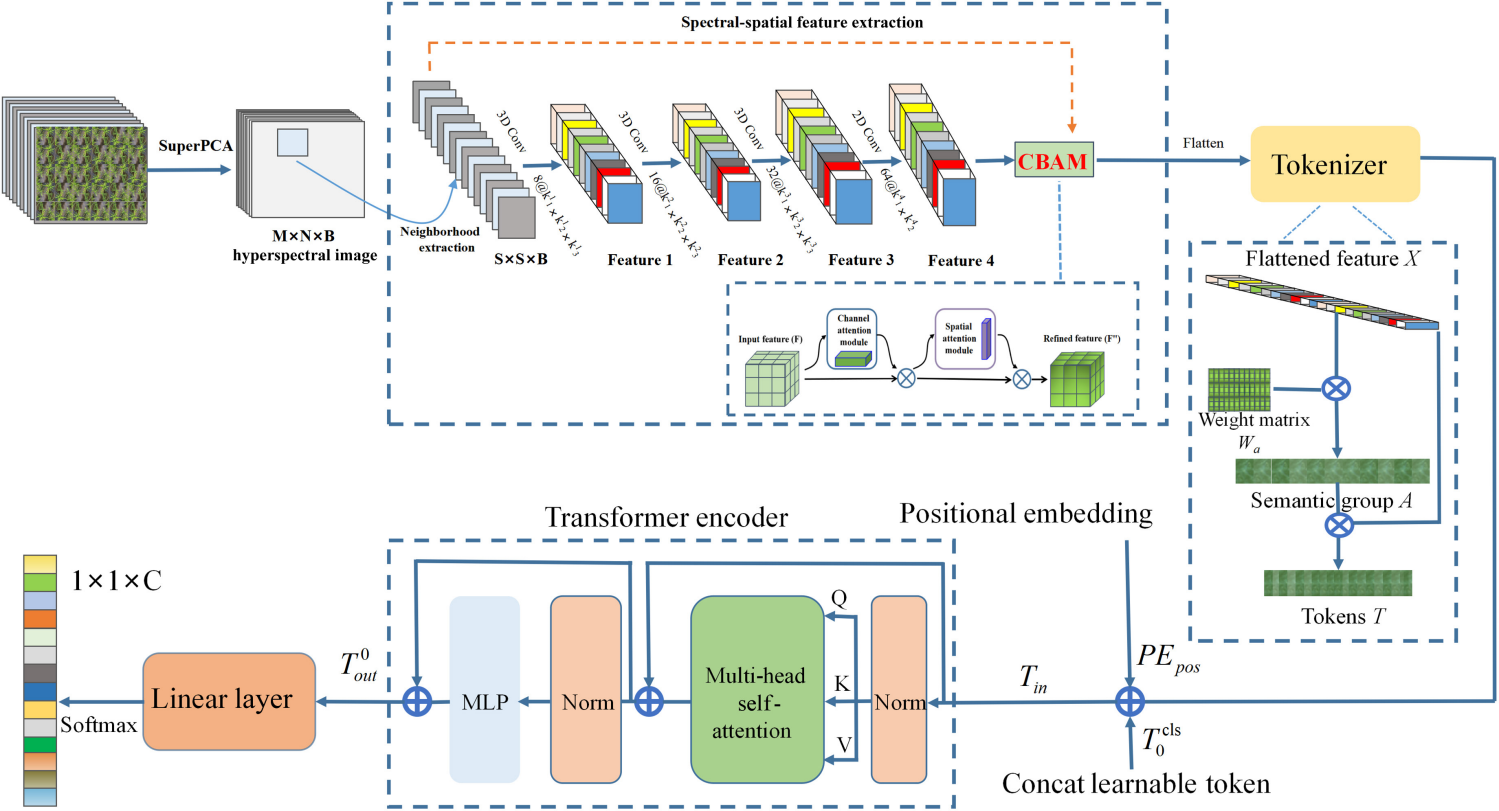
Proposed SHCFTT architecture for modal identification of nutrient stress in rice.

In this work, an HSI-based deep learning framework named SHCFTT was developed to train and verify the recognition of rice stress modes. Figure 3 depicts the configuration of the network. The system primarily has 4 components: an unsupervised feature extraction module, a spectral–spatial feature extraction module, a Gaussian weighted feature tokenizer, and a TE module.

1. Unsupervised feature extraction module

The original rice stress HSI image *I* ∈ *R*^*M*×*N*×*D*^ is used as input, where *M* is the width, *N* is the height, and *D* is the spectral frequency band/depth. Each pixel in *I* has *D* spectral bands, forming a single category vector *Y* = (y_1_, *y*_2_, …, *y_c_*) ∈ *R*^1×1×*Z*^. Here, *Z* is the number of nutrient stress categories in rice. As a result, the D-bands that make up HSI include valuable spectral information. However, it also increases important computational complexity. In order to reduce computational complexity and dimensionality, we introduced an unsupervised feature extraction method based on superpixelwise PCA (SuperPCA) proposed by Jiang et al. [50]. SuperPCA first divided an HSI into multiple uniform regions by superpixel segmentation to obtain the first principal component *I_f_* that captured the most HSI information. Then, entropy rate super-pixel segmentation (ERS) was performed on *I_f_* to achieve superpixel segmentation. Finally, these low-dimensional matrices were rearranged and combined to form a reduced-dimensional hyperspectral image. Due to the use of the ERS method, when it comes to efficacy and efficiency, it performs better than conventional PCA-based overall images. SuperPCA reduces the number of bands from *D* to *B*, leaving the spatial dimension intact. Therefore, we used *X* ∈ *R*^*M*×*N*×*B*^ to represent the SuperPCA reduction data cube, where *X* is the modified input after SuperPCA processing and *B* is the spectral frequency band after SuperPCA processing.

2. Spectral–spatial feature extraction module

The reduced HSI data cube processed by SuperPCA was divided into small overlapping 3D blocks as input data for the model, with the actual label ascertained from the central pixel’s label. The 3D neighboring patches *P* ∈ *R*^*S*×*S*×*B*^ from *X* were created, which were centered around the spatial position (*α*, *β*) and covered the *S* × *S* window/spatial range and all B-bands. Edge pixels are not retrievable when extracting patches encircling a single pixel; these pixels were thus subjected to filling procedures. The width of the filling was (*S*−1)/2. Therefore, the final quantity of 3D neighboring patches generated by SuperPCA was determined by the given *M* × *N*. The width of each patch varied from *α* − (*S* − 1)/2 to *α* + (*S* − 1)/2 and height from *β* − (*S* − 1)/2 to *β* + (*S* − 1)/2.

Three 3-dimensional convolution layers, a 2-dimensional convolution layer, and a convolutional block attention module (CBAM) could extract the spectral spatial features of each 3D neighboring patch. The 3-dimensional convolution layer’s input data were each training sample patch, which had a size of *S*×*S*×*B*. In a 3-dimensional convolutional layer, the calculated value of the *j*th feature cube of the *i*th layer at spatial position (*α*, *β, γ*) was determined by:

$$\upsilon_{i,j}^{\alpha ,\beta ,\gamma }=\Phi \left(\sum_{k}\sum_{h=0}^{H_{i}-1}\sum_{w=0}^{W_{i}-1}\sum_{r=0}^{R_{i}-1}\omega_{i,j,k}^{h^{,},w^{,},r^{,}}\upsilon_{i-1,k}^{a+h^{,},\beta +w^{,},\gamma +r^{,}}+b_{i,j}\right)$$ (5)

Here, Φ(⋅) represents the activation function and *k* represents the characteristic cube associated with the *j*th characteristic cube in the (*i* − 1) layer. The width, height, and spectral dimension of the 3-dimensional convolutional kernel are represented as *H_i_*, *W_i_* and *R_i_*, respectively. $\omega_{i,j,k}^{h^{,},w^{,},r^{,}}$ represents the weight parameter of the position (*h*^,^, *w*^,^, *r*^,^) related to the *k*th feature cube. The *b_i,j_* are the deviations.

The number of convolution kernels in the 3-dimensional convolutional layer was determined in a ratio of 8, 16, and 32. In order to preserve the information from the preceding layer, the number of convolution kernels in the subsequent layer was doubled compared to the number in the layer above. The dimensions of the convolution kernel were 3 × 3 × 7, 3 × 3 × 5, and 3 × 3 × 3, representing, i.e., 2 spatial dimensions and 1 spectral dimension. The number of 2D convolution nuclei was 64, and each nucleus had a size of 3 × 3, representing 2 spatial dimensions. Employing a diminutive convolution kernel can mitigate the superfluous depletion of input information. To increase the number of spectral–spatial feature maps and preserve the spectrum information from the input HSI data in the resultant volume, 3D convolutions were utilized 3 times. Performing a 2-dimensional convolution before the flattened layer for HSI data is critical because a substantial quantity of spectrum information is retained while successfully differentiating spatial information within distinct spectral bands. The CBAM attention module was employed to enhance spatial features by selectively emphasizing key input information and reducing irrelevant regional responses. It was positioned in between the fully connected layer and the 2-dimensional convolutional layer. This technique can be viewed as a dynamic process of selecting crucial input information.

3. Gaussian weighted feature tokenizer

The rice stress features extracted after multiple convolution operations consisted of spectral and spatial information. The Gaussian weighted feature tokenizer was used to further define the feature mapping as semantic markers. It can encode and interpret high-level semantic notions of rice stress HSI feature categories. For this part, the input flat feature map is defined as *G* ∈ *R*^u*v*×*z*^, where *u* is the height, *v* represents the width, and *z* represents the number of channels. Feature tokens are defined as *T* ∈ *R*^*w*×*z*^, where *w* is the number of tokens.

For the feature graph *G*, *T* can be calculated using the following formula:

$$T=\underset{A}{\underbrace{\text{softmax}{\left({\mathrm{GW}}_{a}\right)}^{T}X.}}$$ (6)

where *GW_a_* is the execution of their 1×1 dot-by-dot product and *W*_a_ ∈ *R*^*z*×*w*^ is the weight matrix initialized with a Gaussian distribution. The aim is to map *G* to semantic groups. The size of the semantic group created in this phase is *A* ∈ *R*^u*v*×*w*^. Comparatively important semantic components of *A* are highlighted using softmax(⋅) after A has been transposed. Finally, *T* semantic tokens are produced by multiplying *A* and *G*. Figure 3 illustrates an example of the conversion process.

4. TE module

To understand the connections between advanced semantic features generated by the Gaussian weighted feature tokenizer, the TE module [36] was used. This module first used position embedding to label the location information of each semantic token. Then, the location information *PE_pos_* was encoded and attached to the token representation. The generated embedded semantic token sequence was as follows:

$$T_{0}=\left[T_{0}^{cls},T_{1},\ldots ,T_{w}\right]+PE_{POS}$$ (7)

Here, each token is represented by [*T*_1_, *T*_2_, …, *T_w_*]. The token was connected to a learnable classification token $T_{0}^{\mathrm{cls}}$ that was used to perform the classification task.

*T_in_* was processed through a TE module containing a multihead SA (MSA) block (Fig. 3) and an MLP layer (MSA block and MLP layer with Residual skip connections). Two normalization layers were used to express the deep relationship between modeling semantic markers while having the same size input and output of this module. Among them, the SA (self-attention) mechanism used by the transformer structure can effectively extract the correlation between stress feature sequences.

Each token underwent a linear transformation using 3 weight matrices (*WQ*, *WK*, and *WV*). These matrices were partitioned into 3 parts to construct a 3D invariant matrix for each token. The components of the system consisted of 3 vectors: query (*Q*), key (*K*), and vector (*V*). The attention score for a certain token in SA was computed by taking the dot product of the *K* vector and *Q* vector of the tokens. The score’s weight was determined by the process of normalizing the softmax function. MSA blocks utilize multiple sets of weight matrices in the *Q*, *K*, and *V* maps. The multihead attention value was computed using the identical operating procedure. Subsequently, combine the attention outcomes of each head, as depicted below:

$$\begin{array}{c} \mathrm{MSA}\left(Q,K,V\right)=\text{Concat}\left({\mathrm{SA}}_{1},{\mathrm{SA}}_{2},.\ldots ,{\mathrm{SA}}_{h}\right)W \end{array}$$ (8)

where *h* is the head number, *W* is the parameter matrix, and *W* ∈ *R*^h×*d_K_* ×*d_w_*^, where *d_K_* is the dimension of *K* and *d_w_* = *w* (number of tokens).

Finally, the trained SHCFTT model was operated on new samples and the classification label vector $T_{\text{out}}^{0}$ was used as the input for the top linear layer of the final classification. By using a linear layer and the softmax function, the probability of each sample subjected to each nutrient stress mode was generated. For instance, a specimen may have a 90% likelihood of matching stress treatment E and an 8% probability of matching stress treatment D. Therefore, each sample was labeled with the most likely nutrient stress name. As a result, each pixel in the image was classified as a correlation nutrient stress treatment.

#### Ablation study

In order to fully demonstrate the superiority of the method and evaluate the interaction between the model component modules, ablation experiments of different component combinations were conducted on the 2021+2022 mixed dataset. In order to highlight the differences of modules, a small sample training strategy was used. Seven combinations were considered. The influence of different module components on the whole model is analyzed from the perspective of classification accuracy. The whole model is divided into 5 parts: SuperPCA, CBAM, 3D+2D CNN architecture, tokenizer, and TE. The specific combinations of all models are listed in Table 1. The ablation experiment of the proposed methods was implemented in Python (3.6.5).

**Table 1.** Improved model of ablation experiment

Module	Improved models						
	Case 1	Case 2	Case 3	Case 4	Case 5	Case 6	SHCFTT
SuperPCA	√	—	—	√	√	√	√
CBAM	—	√	—	—	√	—	√
3D+2D CNN architecture	√	√	√	√	√	—	√
Tokenizer	√	√	√	—	—	√	√
TE	√	√	√	—	—	√	√

#### A classification model for comparison (SVM, 1D-CNN, and 3D-CNN)

To evaluate the effectiveness of this method, we adopted a strategy of training and testing on the 2021 dataset, the 2022 dataset, and the combined dataset of 2021 and 2022. The prediction results of the SHCFTT model were compared with those of the classical algorithms using spectral information modeling (SVM, 1D-CNN) and spatial spectral information modeling (3D-CNN) [51]. Seventy percent and 30% of the data were randomly divided into training and testing groups, while for small sample training scenarios, 5% and 95% of the data were randomly divided into training and testing groups, respectively. Additionally, 10-fold cross-validation was used in this study to assess the performance of the model. Every experiment was carried out on a Lenovo Thinkpad P15V laptop with 16 GB of RAM and an NVIDIA Quadro P620 graphics processing unit (GPU).

### Model assessment

The effectiveness of the model in identifying nutrient stress in rice was assessed in this study using 3 evaluation indicators: overall accuracy (OA), average accuracy (AA), and Kappa coefficient (Kappa). The ratio of accurately classified samples to total samples is represented by the OA. The AA is the ratio of the total number of samples in each class divided by the number of correctly predicted samples. The AA for every class was taken into account at the end. A statistical metric called kappa can be used to determine how well the truth map and the classification map agree with one another.

$$\mathrm{OA}=\frac{\sum_{i=1}^{n}\;x_{\mathrm{ii}}}{\sum_{i=1}^{n}\;\sum_{j=1}^{n}\;x_{\mathrm{ij}}}$$ (9)

$$\mathrm{AA}=\frac{1}{n}\sum_{i=1}^{n}\frac{x_{\mathrm{ii}}}{\sum_{j=1}^{n}x_{\mathrm{ij}}}$$ (10)

$$\begin{array}{c} \text{Kappa}=\frac{\sum_{i=1}^{n}\sum_{j=1}^{n}x_{\mathrm{ij}}\times \sum_{i=1}^{n}x_{\mathrm{ii}}-\sum_{\begin{array}{c} i=1\\ i=j \end{array}}^{n}\left(\sum_{i=1}^{n}x_{\mathrm{ij}}\times \sum_{j=1}^{n}x_{\mathrm{ij}}\right)}{{\left(\sum_{i=1}^{n}\sum_{j=1}^{n}x_{\mathrm{ij}}\right)}^{2}-\sum_{\begin{array}{l} i=1\\ i=j \end{array}}^{n}\left(\sum_{i=1}^{n}x_{\mathrm{ij}}\times \sum_{j=1}^{n}x_{\mathrm{ij}}\right)} \end{array}$$ (11)

In the formula, *i* = 1, 2, 3, ⋯, *n* represents the number of categories of real rice stress samples, *j* = 1, 2, 3, ⋯, *n* represents the number of categories for predicting rice stress samples, *n* represents the total number of stress mode categories, *x_ij_* represents the number of class *i* samples, but predicted as class *j*, and *x_ii_* represents the number of samples with both real and predicted classes *i*.

## Results

### Spectral diversity of rice under different nutrient stress

**Fig. 4.**
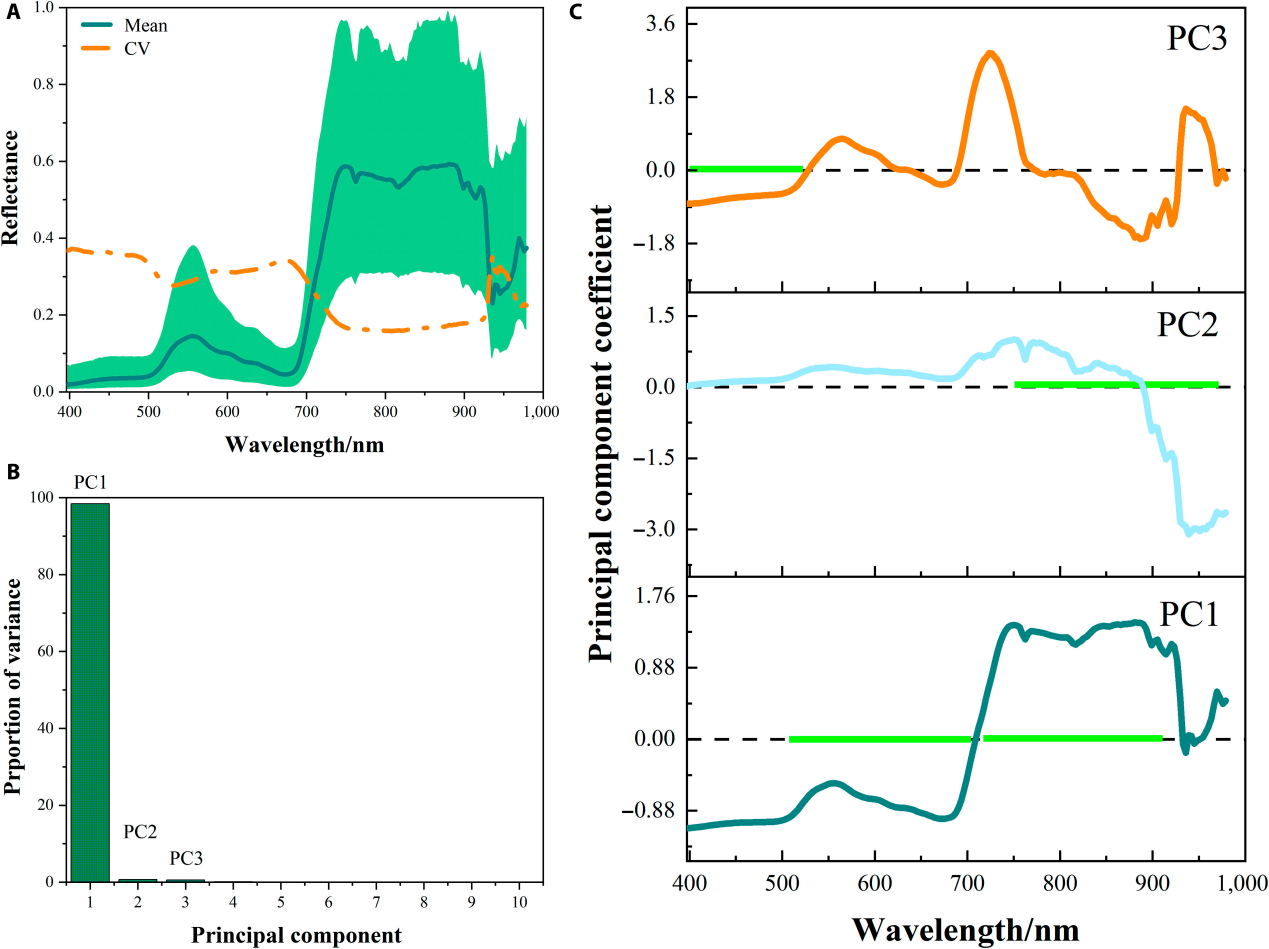
Spectral reflectance and PCA of all datasets. (A) Average spectral reflectance and coefficient of variation (CV) of all datasets. (B) The first 10 principal components of rice reflectance under nutrient stress. (C) The principal component coefficient of the first 3 PCs with the horizontal green line indicates spectral regions with the highest proportion of variance of rice reflectance.

**Fig. 5.**
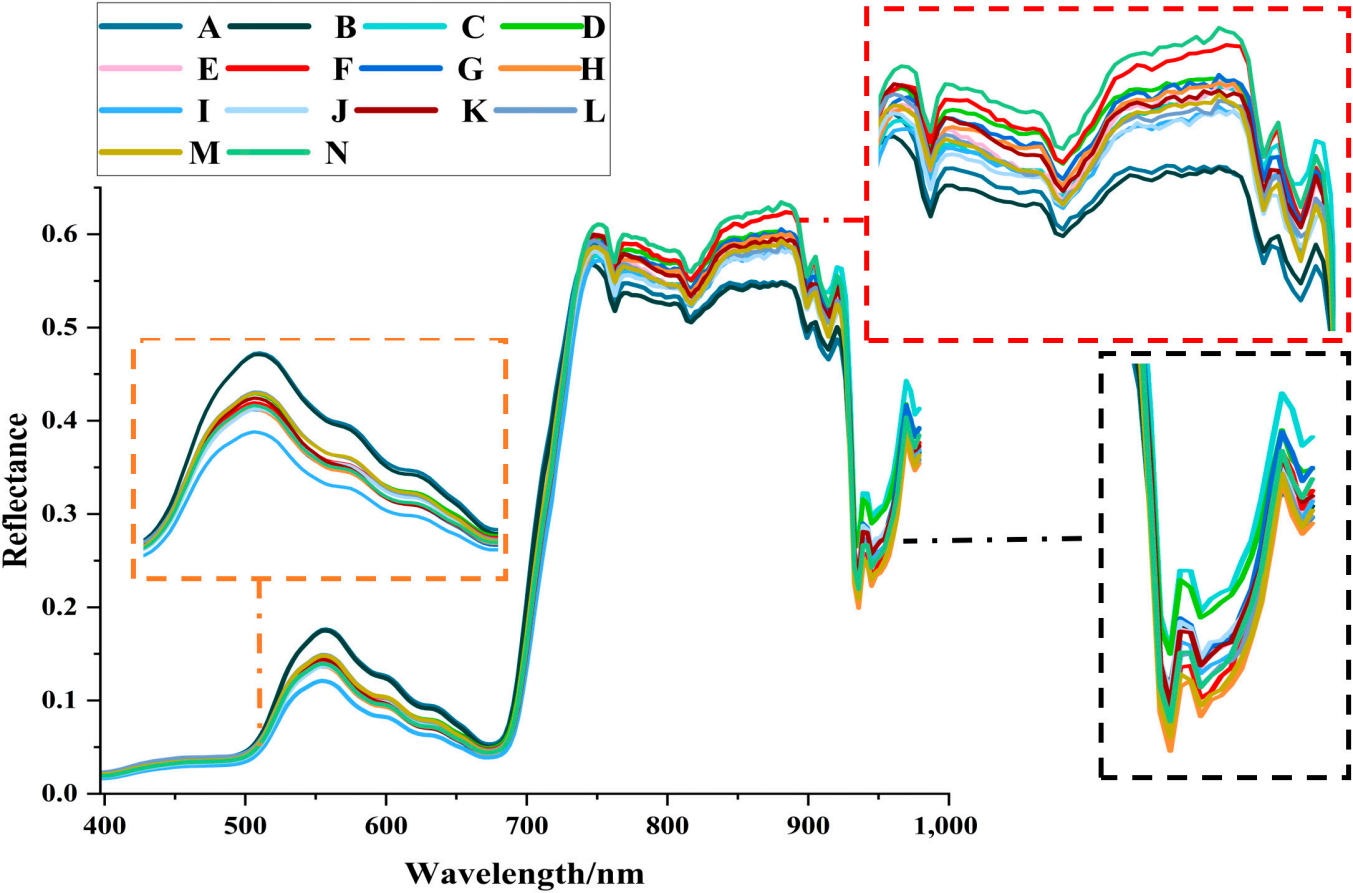
Average spectral curves of rice under different nutrient stress.

The alterations in the optical properties of the reflectance spectrum of rice leaves are related to the subtle structural features and specific modifications of biochemical elements within the leaves [52]. HSI can reveal a subtle shift in physiological data following stress as depicted in Figs. 4 and 5. While stressed and unstressed rice plants show distinct spectral features, the general trend of the spectral curves is the same. In the visible green light region (520 to 600 nm), rice plants have a lower photosynthetic effect, less light absorption, and a higher reflectance at 550 nm, which results in the formation of a reflection peak, also referred to as a “green peak” [53]. At 680 nm, in the visible red light region (630 to 690 nm), a reflection valley known as the “red valley” forms. This is the spectral band where plants have the strongest chlorophyll absorption and photosynthetic activity while having more light absorption and lower reflectivity. The hyperspectral curve rises quickly in the 700- to 930-nm NIR range, reaching its maximum point at 760 nm while forming a reflection platform. Mesophyll thickness, density, and stomatal structure are among the anatomical features that affect the internal light scattering of the leaves, which in turn affects the reflectivity of this region [54].

After collecting on-site data, the spectral reflectance curves of rice under various nutrient stressors were extracted and compared in Fig. 5. Figure 5 shows the curves related to the spectral profiles of rice including the reference treated F (N_2_P_2_K_2_) and samples under extreme as well as compound nutrient stress. The spectrum responses of leaves varied under different nutritional stressors, resulting in distinct spectral profiles. During periods of nutritional stress, the leaves of A (N_0_P_0_K_2_) and B (N_0_P_2_K_2_) exhibited increased reflectivity in the green and red regions of the electromagnetic spectrum. This increase in reflectance suggests a decline in the photosynthetic process and a reduced capacity of rice plants to absorb red light. The stress treatments A and B exhibited the least amount of reflectance in the 740- to 900-nm range, indicating the potential breakdown of the mesophyll structure in rice leaves. The stress treatments A and B exhibited the most minimal reflectance throughout the 740- to 900-nm range, indicating the presumed degradation of the mesophyll structure in rice leaves. Similarly, the spectral profiles of rice canopies exhibited distinct spectral responses when subjected to various nutrient pressures. The alterations in the visible light spectral profile, pertaining to health, can be regarded as either an augmentation or reduction in photosynthesis; this suggests that modifications in leaf characteristics resulting from nutrient stress can be identified and examined by observing variations in visible light reflectance. Furthermore, the reduction in photosynthetic activity can also be shown by a drop in the fluorescence energy around the wavelength of 760 nm. Chlorophyll absorbs solar energy and utilizes it for carbon fixation and heat dissipation, then releases longer wavelength emittance sources in the form of chlorophyll fluorescence [55]. The third stage of photosynthesis involves chlorophyll fluorescence, when plants emit low-energy photons that can provide information into their overall health. Except for stress treatment N (N_2_P_1_K_1_), other stress treatments showed an apparent decrease near 760-nm wavelength compared to baseline treatment F (N_2_P_2_K_2_), indicating a reduction in fluorescence release due to the decline of photosynthesis. On the other hand, the NIR region of all nutrient treatment groups showed a sharp decrease and increase as well as very subtle changes. The above changes are related to the characteristics of NPK. A nitrogen deficiency in leaf cell proteins usually leads to a decrease in both leaf chlorophyll concentration and dry matter protein content [56], due to the substantial allocation of nitrogen in these proteins. Insufficient phosphorus levels have an adverse effect on both cell division and growth in plants, as well as the number of cells per unit of leaf area [57]. K is responsible for initiating and/or improving many enzymes that affect the levels of sugar and starch in plants [58]. These different biochemical and physiological changes are related to different nutritional stresses, which may lead to subtle differences in canopy spectra.

The spectral diversity of leaves reflects the trend of changes in leaf reflectance under different nutrient stress types and levels. The PCA of all rice nutrient stress data showed that the first 3 principal components (pc) represented 99.72% of the sample reflectance variance (Fig. 4B). Figure 4C further illustrates the spectral regions related to the first 3 PCs. PC1 and PC2 dominated the main changes in rice leaf reflectance in the visible and NIR regions. VIS spectra were related to the vibration, winding, and twisting of O-H, C-H and N-H, respectively. The changes in NIR spectra reflected the stretching vibration of the third overtones of O-H and C-H of rice leaf structure and water [59]. PC1 was negative before 710 nm and then positive except for the area near 940 nm, having a larger amplitude in the NIR region. PC2 was negative after 880 nm. The main spectral characteristics of PC3 corresponded to the visible blue–purple light region in the 400- to 520-nm region, which was related to the auxiliary processes of photosynthesis such as lutein and carotenoids [17].

**Fig. 6.**
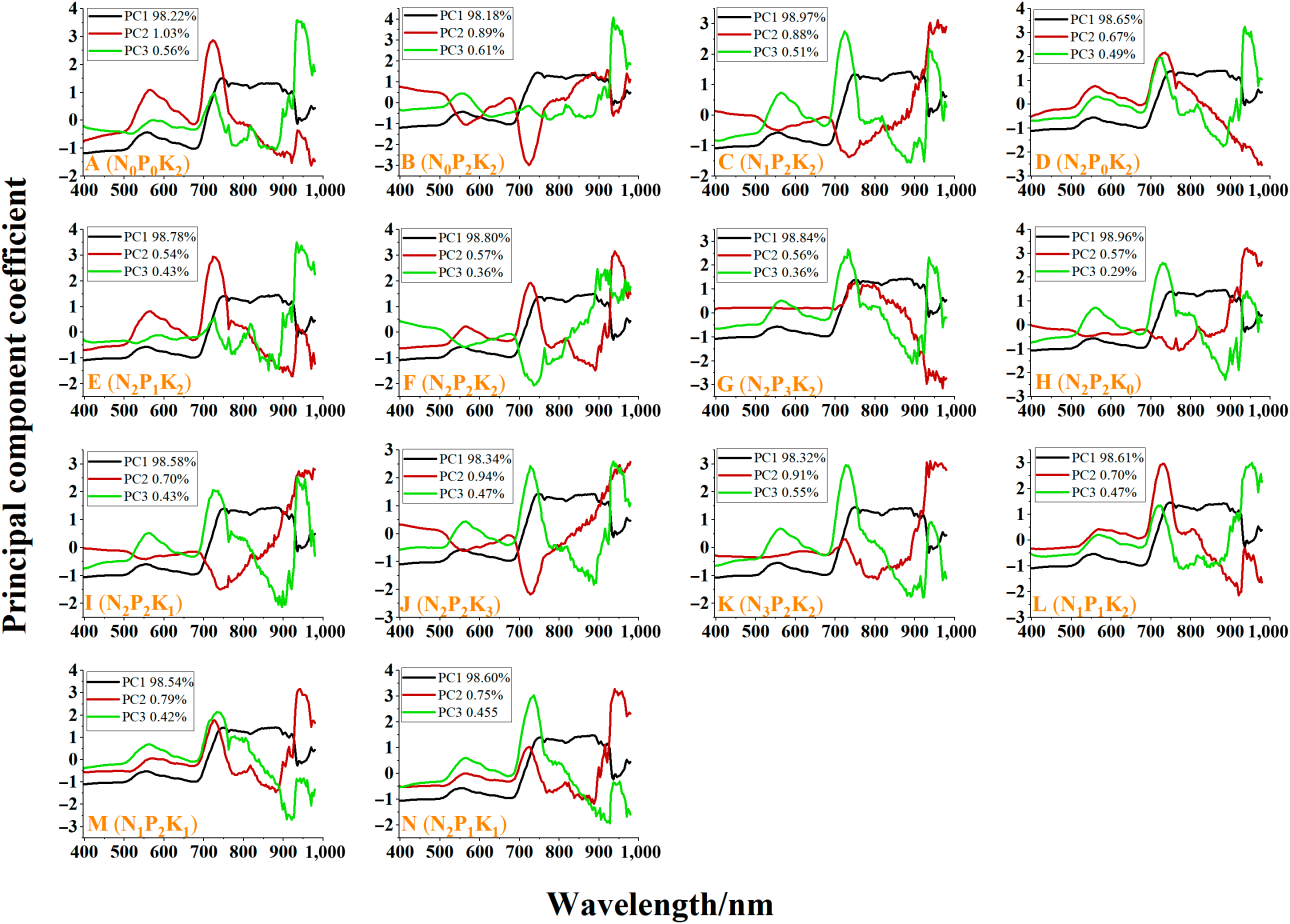
The first 3 principal components (PCs) explain the highest variance ratios for different spectral regions obtained from different nutrient stress types and levels. (A) to (N) represent different kinds of stress treatment: A (N_0_P_0_K_2_), B (N_0_P_2_K_2_), C (N_1_P_2_K_2_), D (N_2_P_0_K_2_), E (N_2_P_1_K_2_), F (N_2_P_2_K_2_), G (N_2_P_3_K_2_), H (N_2_P_2_K_0_), I (N_2_P_2_K_1_), J (N_2_P_2_K_3_), K (N_3_P_2_K_2_), L (N_1_P_1_K_2_), M (N_1_P_2_K_1_), and N (N_2_P_1_K_1_).

Specifically, leaf reflectance from NPK single or compound stress from stress treatment A (N_0_P_0_K_2_) to stress treatment N (N_2_P_1_K_1_) exhibited extremely complex patterns on the 3 PCs (Fig. 6). Figure 6B (N_0_P_2_K_2_), C (N_1_P_2_K_2_), F (N_2_P_2_K_2_), and K (N_3_P_2_K_2_) show the changes in leaf reflectance in response from 0 levels to 3 level N stress. Different N stresses showed similar PC1, but PC2 and PC3 showed different patterns, especially at around 550nm and in the VIS region. Figure 6D (N_2_P_0_K_2_), E(N_2_P_1_K_2_), F(N_2_P_2_K_2_), and G(N_2_P_3_K_2_), and H (N_2_P_2_K_0_), I(N_2_P_2_K_1_), F(N_2_P_2_K_2_), and J(N_2_P_2_K_3_) show the spectral diversity of P and K stressed leaves from 0 level to 3 levels, respectively. P stress showed a similar pattern in the 750- to 900-nm region of PC2 and K stress showed a similar pattern in the 900- to 950-nm region of PC2. In addition, PK stress showed a similar pattern in the 900- to 1000-nm region of PC3 but different patterns in other regions. Leaf spectral diversity also varied with stress types. Figure 6B (N_0_P_2_K_2_), D (N_2_P_0_K_2_), and H (N_2_P_2_K_0_) exhibited similar patterns in the 398- to 700-nm region at PC3, but exhibited different patterns at PC2. The proportion of variation covered by the first 3 pcs differed according to the type and extent of nutrient stress, with PC1 accounting for 98.18% to 98.97% of the variation in spectral reflectance due to the adoption of the same type of rice cultivars with similar proportions of variance. PC2 and PC3 covered only a small part of the spectral reflectance variance (0.54% to 1.03% and 0.29% to 0.61%) but reflected the diversity of spectra from different stressed leaves. It might be related to some subtle absorption properties of proteins and tissue structures induced by NPK biochemical activities.

### Vegetation index analysis

**Fig. 7.**
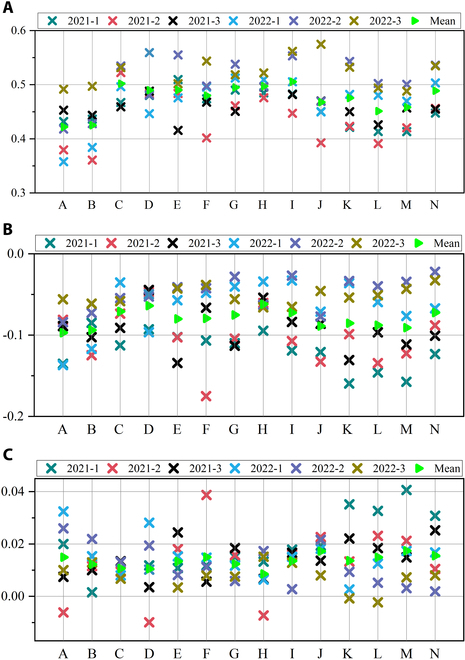
Three vegetation indices of rice under different nutrient stress. (A) Normalized difference vegetation index (NDVI). (B) Photochemical reflectance index (PRI). (C) Plant senescence reflectance index (PSRI). For 14 nutrient stress treatments, the average vegetation index (represented by a green triangle) comes from all samples under one stress treatment. The *x*-axis label displays the stress treatment and the *y*-axis represents NDVI, PRI, and PSRI.

The hyperspectral images collected by SPECIM IQ can provide a rich spectral message, especially in the visible and NIR spectral regions. Apart from the 204 spectral bands of HSI, 3 vegetation indices were calculated to heighten the spectral feature space and study stress patterns by combining information from different bands. Figure 7 shows a representative pattern of changes in rice plants under different nutrient stresses over a 2-year period using NDVI, PRI, and PSRI. For example, Fig. 7B (N_0_P_2_K_2_), C (N_1_P_2_K_2_), F (N_2_P_2_K_2_), and K (N_3_P_2_K_2_) show an NDVI trend of N stress from 0 to 3 levels, with treatment B being the lowest, treatment C reaching its peak, and treatment F as well as treatment K both being similar in the middle position. Stress treatment D (N_2_P_0_K_2_), E (N_2_P_1_K_2_), F (N_2_P_2_K_2_), and G (N_2_P_3_K_2_) showed an NDVI trend from 0 to 3 levels of P stress, with the exception of treatment F, which was the lowest; the other 3 levels showed similar values. The stress treatment H (N_2_P_2_K_0_), I (N_2_P_2_K_1_), F (N_2_P_2_K_2_), and J (N_2_P_2_K_3_) showed an NDVI trend from 0 to 3 levels of K stress, with NDVI values sorted as F < G < H < I. As the degree of K stress increased, the PRI continued to decrease for the PRI. In addition, PSRI also had a good indicator for K stress. The PSRI index continued to rise with the increase in the degree of stress. During the growth stage of rice, almost all stress-treated rice leaves exhibited high reflectance in the upper part of the canopy, making it difficult to distinguish between diverse stresses. However, the upper canopy spectrum of rice varied due to different types and degrees of nutrient treatment, which created the possibility of distinguishing different nutrient stress. In addition, during the growth period of rice, the lower part of the canopy and the root system are hidden in the water. Therefore, the spectral response of rice under different nutrient stresses is mainly composed of the upper canopy and exhibits differences between different nutrient stresses.

### Unsupervised data visualization

**Fig. 8.**
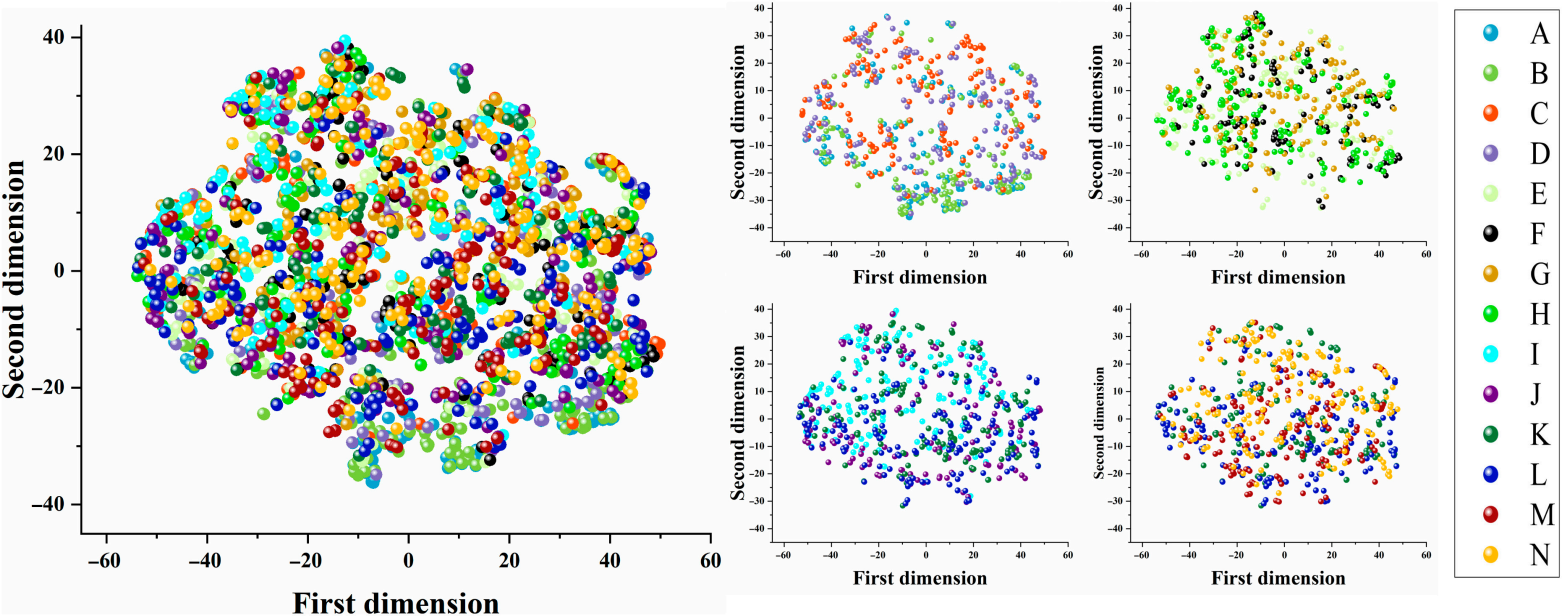
Unsupervised visualization of rice spectral information under 14 nutrient stress treatments using t-SNE.

Fourteen clusters were identified in the rice nutrient stress spectrum, each of which was assigned a stress label. In order to comprehend variations in spectral response, the unsupervised visualization process looked more closely at natural patterns between sample data. Figure 8 shows a complex cluster scenario. In line with the data shown in the previously mentioned original spectral map, all of the stressed sample’s clusters were extremely close to one another and poorly separated. The similarity of fingerprints under various stress conditions could be the cause. The spectral data of rice leaves under various nutrient stresses showed varying degrees of correlation and overlapped information, which could potentially impede the classification process. Furthermore, disparities were observed in the nutrient stress data, which could be explained by a variety of factors, including the structural alterations in leaves brought about by various NPKs, the “fingerprint” features of the samples, and the great degree of variability found in natural samples. These data visualization graphs have demonstrated, on the whole, the separability and aggregation pattern of rice data under various nutrient stresses. It is challenging to fully differentiate between them using the original spectrum alone, though, regardless of the type of stress. As such, identification via end-to-end modeling is required.

### Ablation experiment results

Models without 3D+2D CNN architecture and CBAM have the worst classification accuracy (Case 6). In the absence of SuperPCA and CBAM, this model (Case 3) outperforms the first Case (Case 6). On the basis of Case 3, by adding Case 2 of the CBAM module, the classification accuracy AA is increased by 1.54%. The comparison of Case 1 and Case 4 models shows that using Tokenizer and TE modules, the classification accuracy of the model is improved by 3.86%. The comparison between the Case 2 model and SHCFTT shows that SuperPCA, the unsupervised feature extraction module, improves the classification accuracy of the model by 6.65%. Compared with Case 1 and Case 6, the role of 3D+2D CNN architecture in the spectrum-space feature extraction module is highlighted, and the classification accuracy of the model increases by 11.1%, which indicates that the CNN architecture contributes to the improvement of performance. Case 1 achieves a classification accuracy of 93.17%, which is considered relatively good, but this accuracy is slightly lower than our proposed method. In summary, the analysis of the ablation results (Table 2) further confirms the validity of the proposed SHCFTT model and clearly demonstrates the role and function of these key modules.

**Table 2.** Results of ablation experiment

Methods	Indicators		
	Average accuracy (%)	Overall accuracy (%)	Kappa coefficient (×100)
Case 1	93.17	93.19	92.66
Case 2	87.44	87.41	86.44
Case 3	85.90	85.86	84.78
Case 4	89.31	89.32	88.50
Case 5	90.04	90.02	89.25
Case 6	82.07	82.07	80.69
SHCFTT	94.09	94.08	93.94

### A deep learning classification network for identifying nutrient stress in rice

The test 8 nutrient stress identification models were trained for each round of sampled dataset. By combining nutrient stress datasets from different years (2021+2022), the other 4 models were trained and tested. The OA, AA, and kappa coefficient were introduced to evaluate the performance of the model. The larger the values of each indicator, the better the identification effect. Table 3 presented the rice stress recognition results of SVM and 1D-CNN models trained using spectral data as well as 3D-CNN and SHCFTT models trained using spatial spectral data. All models had shown very high accuracy in training and testing single-year datasets. The overall training accuracy OA of a single model was between 99.54% and 100%. The overall validation accuracy OA was between 89.85% and 100%. The OA, AA, and Kappa coefficient of the SHCFTT model in the single-year datasets were all 100%. The performance of the developed rice nutrient stress deep learning recognition model was evaluated using hyperspectral images from a biennial dataset (2021+2022). The OA of all classification models was above 90.21%. The AA was above 90.20% and the Kappa coefficient was above 89.46. This indicates that the classical classification model can accurately identify rice with different stress levels. However, the SHCFTT model designed in this article had higher recognition accuracy than other models in the biennial dataset while having 100% OA, AA, and Kappa coefficient. This indicates the positive role of the proposed model architecture in identifying nutrient stress in rice.

**Table 3.** Results of rice nutrient stress identification model

		Training set			Testing set		
		Average accuracy (%)	Overall accuracy (%)	Kappa coefficient (×100)	Average accuracy (%)	Overall accuracy (%)	Kappa coefficient (×100)
SVM	2021	99.82	99.82	99.82	89.81	89.85	89.07
	2022	100.00	100.00	100.00	90.90	90.25	89.50
	2021+2022	99.54	99.54	99.51	90.20	90.21	89.46
1D-CNN	2021	99.94	99.94	99.94	93.30	93.32	92.81
	2022	99.88	99.88	99.88	94.11	94.12	93.67
	2021+2022	99.88	99.88	99.88	92.87	92.92	92.38
3D-CNN	2021	100.00	100.00	100.00	97.78	97.76	97.60
	2022	100.00	100.00	100.00	97.32	98.28	97.08
	2021+2022	100.00	100.00	100.00	96.33	96.33	96.05
SHCFTT	2021	100.00	100.00	100.00	100.00	100.00	100.00
	2022	100.00	100.00	100.00	100.00	100.00	100.00
	2021+2022	100.00	100.00	100.00	100.00	100.00	100.00

### Identification results under small training samples

Most of the practical problems can now be solved using machine learning techniques, especially deep learning techniques, as long as the available data adequately represents the changes taking place in practice. Therefore, the major bottleneck in utility development is the size and quality of the dataset used to train and test the model [60]. In general, the data available are too limited to generate robust models. Moreover, supervised learning requires proper labeling of these data. In agriculture, the task is even more challenging because of the harsh field environment, test cycles, and costs. Moreover, visual markers of interest are not always present. Controlled trials can minimize this problem by limiting the presence of empty spectrum signals of interest induced by nutrient inputs. However, few labeled instances still pose substantial challenges in practical scenarios. Therefore, we discussed the results of classification models with small training samples, where the training samples only account for 5% of the total samples and summarized the results in Table 4. At this point, the classification results of all models in a single dataset showed varying degrees of decline, but surprisingly, the OA of the developed SHCFTT model was over 93.92%, the AA was over 93.93%, and the Kappa coefficient was above 93.77%. The accuracy rates of SVM, 1D-CNN, 3D-CNN, and SHCFTT in the 2-year dataset (2021+2022) were 35.99%, 28.01%, 71.73%, and 94.08%, respectively. From the experimental results, it can be seen that with the sharp decrease in training data, the performance of each model decreased, whether in a single dataset or a 2-year dataset. Even with extremely limited training samples, SHCFTT can still achieve identification accuracy that exceeds the comparison method. This proves the effectiveness and robustness of the proposed SHCFTT in this article under small sample conditions. It will be useful for its application in practical scenarios.

**Table 4.** Identification results under small training samples

		Training set			Testing set		
		Average accuracy (%)	Overall accuracy (%)	Kappa coefficient (×100)	Average accuracy (%)	Overall accuracy (%)	Kappa coefficient (×100)
SVM	2021	100.00	100.00	100.00	37.81	37.80	33.02
	2022	100.00	100.00	100.00	37.17	37.15	32.32
	2021+2022	100.00	100.00	100.00	35.99	35.99	31.06
1D-CNN	2021	100.00	100.00	100.00	31.01	30.99	25.68
	2022	100.00	100.00	100.00	34.35	34.37	29.32
	2021+2022	100.00	100.00	100.00	28.02	28.01	22.48
3D-CNN	2021	100.00	100.00	100.00	74.79	74.73	72.79
	2022	100.00	100.00	100.00	75.25	75.20	73.30
	2021+2022	100.00	100.00	100.00	71.75	71.73	69.56
SHCFTT	2021	100.00	100.00	100.00	93.93	93.92	93.77
	2022	100.00	100.00	100.00	94.32	94.28	94.08
	2021+2022	100.00	100.00	100.00	94.09	94.08	93.94

## Discussion

### Spectral diversity of rice leaves under nutrient stress

The spectral properties of plants change with various nutrient stresses and levels. In agricultural environments, variations in vegetation phenology and productivity are driven by the shedding of old leaves and the emergence of new leaves while the specificity of photosynthesis is explained by the growth of leaves and the accumulation of matrix elements [61]. Most nutritional stresses lead to apparent chlorosis [62–64], resulting in similar spectral characteristics. Masoni et al. [65] examined the effects of iron (Fe), sulfur (S), magnesium (Mg), and manganese (Mn) deficiency on leaf reflectance of barley, wheat, corn, and sunflower. They found that nutrient shortage lowered Chl concentration in all species, resulting in an increase in reflectance between 400 and 1,100 nm and a shortened red edge location. This makes it tough to differentiate distinct nutritional stress states using canopy spectra. Nevertheless, the biochemical and physiological changes caused by different nutrient stress in crops are different. In Figs. 4, 5, 6, and 7, the spectral profiles, vegetation indices, and PCA data of rice leaves under different nutrient stresses were presented, which showed that the visible light region of 500 to 670 nm and the NIR region were the most prominent areas of rice nutrient stress. They can reflect the spectral diversity of rice nutrient stress leaves and indirectly reflect the physiological activity of rice, which may play a crucial role in identifying rice nutrient stress. In addition to the diversity of rice nutrient stress spectra, the characteristics of nutrient stress spectra may also vary with changes in plants [63,66]. However, the leaf spectrum of plants is a phenotypic expression of the total signal of chemical and structural components generated over time, reflecting the chemical adaptability of plants to environmental conditions, including factors such as climate, nutrient availability, and biological interactions [67]. Therefore, as the external environment or the plant’s internal nutritional pattern changes, spectral variation will also provide greater differentiation ability. High-resolution terrestrial hyperspectral techniques can even extend plant phenotype monitoring from nutrient stress identification to species, function, and gene diversity dimensions [68]. For the purpose of remote agricultural nutrient stress analysis, a thorough grasp of the physiological and biochemical reactions of crops to various nutritional stresses as well as the connection between their spectrum properties, is thought to be essential. Future studies should focus on bridging the gap between sensitive spectral characteristics and possible physiological and biochemical responses of crops to various nutritional stressors.

### SHCFTT model capability for identifying nutrient stress in rice

The classification of HSI is a fundamental analytical task, which has gained a lot of traction. Nevertheless, because of the similarity between high-dimensional features, spectra, and difficulty in obtaining labeled samples, it has been difficult to classify HSI data in an efficient and precise manner for many years. To address these issues, some classic classification models and deep learning classification models have been used. In this work, we provided evidence that the nutrient stress mode of rice can be accurately identified through rapid and non-destructive HSI measurements, and the methods such as spectrum profile, VI, and no supervision and visualization were used as mappings for nutrient stress responses. Three classic classification algorithms were studied and SHCFTT models were developed to determine the relationship between target stress modes, and spectral and spatial features. The OA, AA, and Kappa coefficient of each round of classification indicated that classic hyperspectral classification methods based on spectral information did not utilize the spatial information of hyperspectral images, resulting in limited classification accuracy. Specifically, the classic methods used in this article for identifying rice stress using spectral information, SVM, and 1D-CNN had an OA of less than 94.12% under 70% training sample conditions and less than 37.80% under small sample training conditions. The deep learning classification model 3D-CNN, which utilized the spatial spectral feature information of hyperspectral images, had an OA of over 96.33% and 71.73% under 70% training sample conditions and small sample conditions, respectively. It was compared with advanced plant nutrient stress detection. For example, Anusia et al. [69] used artificial neural network (ANN) to identify nutrient stress in oil palm plants, and the accuracy of the established model for the detection of optimal nitrogen and excess nitrogen in oil palm plants reached 100%. However, the accuracy of this model is only 70% for the classification of nitrogen and potassium deficiency. The SHCFTT model developed in this article was based on a transformer network architecture, and demonstrates the role of each module in SHCFTT network architecture through ablation experiment. In general, the model combines unsupervised feature extraction, spectral–spatial feature extraction based on CNN, and CBAM with transformer-based feature learning. The network’s underlying feature selection was guided by extracting deep-level abstract spectral spatial features, utilizing feature labeling and TE modules for feature learning. A 100% classification accuracy of a single year and biennial datasets was achieved under 70% training sample conditions. The classification accuracy of a single year and biennial datasets was greater than 93.92% under small sample training conditions. This model can distinguish nutrient deficiency/excess from appropriate rice in the early stages before obvious symptoms of nutrient stress appear. In addition, such predictions can be used in the area of global food security, especially in the detection of crop stress in the field. This model can also provide a more reliable way for high-throughput and non-destructive phenotype research.

### Potential applications and future prospects

The SHCFTT model has demonstrated a high level of accuracy in hyperspectral analysis. Consequently, it is expected to yield superior results in recognizing rice nutritional stress compared to the use of multispectral aerial images in rice nutrient stress tests. Multiple investigations, for example, Ferreira et al. and Awad et al. [70,71], used different remote sensing data to map tree species in forests and have shown that the precision of plant mapping has been enhanced by utilizing plants with higher spectral resolution. However, one of the main limitations of the widespread use of hyperspectral for nutrient stress identification is that it is difficult to determine a reliable calibration procedure and spectral algorithm under many growth conditions such as soil type, growth stage, variety, and weather. In the operating environment, remote sensing alone cannot distinguish whether the nutrient status of time and location is related to soil, growth period, weather, variety, and management. Therefore, it is crucial to understand the differences between these factors and develop a universal algorithm, which may be achieved by physiological analysis, crowdsourcing technology, and transfer learning. We could achieve this in the future by developing objective solutions, resource productivity improvements, and serving sustainable agriculture.

### Conclusion

This study utilized terrestrial hyperspectral remote sensing technology to collect a total of 420 hyperspectral images of rice under different nutrient stresses from 2021 to 2022. Spectral profiles, vegetation indices, PCA, and other methods were used as mappings for nutrient stress responses to characterize the spectral diversity and physiological activity of rice leaves under nutrient stress. In addition, a transformer-based deep neural network SHCFTT was developed to identify and classify rice nutrient stress patterns from hyperspectral images. The results were compared with classical SVM, 1D-CNN, and 3D-CNN in different year datasets. Considering the identification of nutrient stress in rice regardless of the modeling strategy, the SHCFTT model always maintained the best predictive performance with an OA ranging from 93.92% to 100%. The proposal of these methods not only has a positive effect on identifying nutrient stress in rice but also has implications for monitoring and decision-making of crop health status in the field and precision agriculture. This study is a typical case of highly diverse nutrient stress in an intense field scenario, contributing to the development of HSI crop phenotype research and precision agriculture field information perception.

## Data Availability

The data used to support the findings of this study are available from the corresponding authors upon request.
