# Supplementary material for: Characterization and Identification of NPK Stress in Rice Using Terrestrial Hyperspectral Images
Source: Plant Phenomics. 2024 Jul 24;6:0197. doi: 10.34133/plantphenomics.0197 (PMC11266478; doi:10.34133/plantphenomics.0197)
Supplement: Supplementary 1 — Supplementary Methods Fig. S1 [file plantphenomics.0197.f1.pdf]

Figure Captions:

Fig. S1. Typified examples of masking processing.

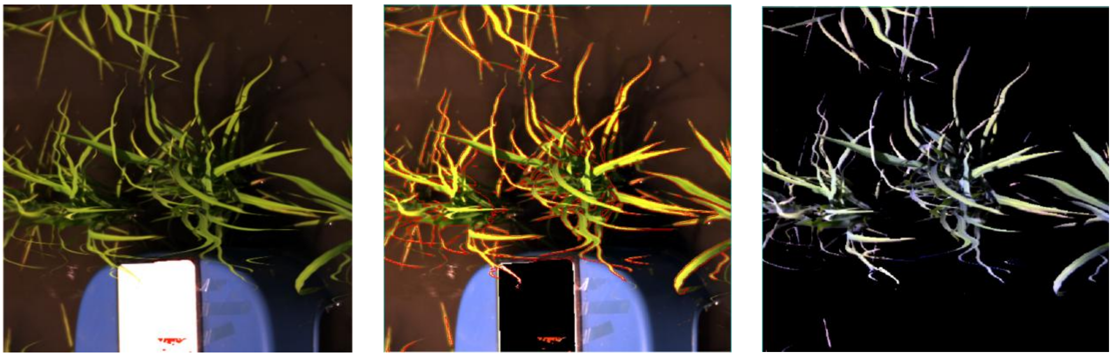

Fig. S1. Typified examples of masking processing.
